# Divergent Traits and Ligand-Binding Properties of the Cytomegalovirus CD48 Gene Family

**DOI:** 10.3390/v12080813

**Published:** 2020-07-28

**Authors:** Pablo Martínez-Vicente, Domènec Farré, Pablo Engel, Ana Angulo

**Affiliations:** 1 Immunology Unit, Department of Biomedical Sciences, Faculty of Medicine and Health Sciences, University of Barcelona, 08036 Barcelona, Spain; dombcn@gmail.com (D.F.); pengel@ub.edu (P.E.); 2 Institut d’Investigacions Biomèdiques August Pi i Sunyer, 08036 Barcelona, Spain

**Keywords:** viral immune evasion, viral evolution, viral homologs of host genes, cytomegalovirus, CD48, 2B4, CD2, T lymphocytes, NK cells

## Abstract

The genesis of gene families by the capture of host genes and their subsequent duplication is a crucial process in the evolution of large DNA viruses. CD48 is a cell surface molecule that interacts via its N-terminal immunoglobulin (Ig) domain with the cell surface receptor 2B4 (CD244), regulating leukocyte cytotoxicity. We previously reported the presence of five CD48 homologs (vCD48s) in two related cytomegaloviruses, and demonstrated that one of them, A43, binds 2B4 and acts as a soluble CD48 decoy receptor impairing NK cell function. Here, we have characterized the rest of these vCD48s. We show that they are highly glycosylated proteins that display remarkably distinct features: divergent biochemical properties, cellular locations, and temporal expression kinetics. In contrast to A43, none of them interacts with 2B4. Consistent with this, molecular modeling of the N-terminal Ig domains of these vCD48s evidences notable changes as compared to CD48, suggesting that they interact with alternative targets. Accordingly, we demonstrate that one of them, S30, tightly binds CD2, a crucial T- and NK-cell adhesion and costimulatory molecule. Thus, our findings show how a key host immune receptor gene captured by a virus can be subsequently remodeled to evolve new immunoevasins with altered binding properties.

## 1. Introduction

Viral infections elicit a strong and broad reaction based on the coordinated action between the innate and adaptive immunity. In these responses, NK cells and T lymphocytes play a critical role in the successful recognition and elimination of virally infected cells [1,2,3]. The efficient activation of NK and T cells entails numerous activating receptors and co-stimulatory molecules [4,5,6,7]. Among them, 2B4 (CD244), a transmembrane protein belonging to the signaling lymphocyte activation molecule (SLAM) family, is crucially implicated in the regulation of leukocyte cytotoxicity and cytokine production [8,9]. It interacts with its natural ligand CD48, another SLAM family member and a glycosylphosphatidylinositol (GPI) anchored molecule, which is expressed on nearly all hematopoietic cells. The binding of CD48 to 2B4 induces the phosphorylation of the cytoplasmic immunoreceptor tyrosine switch motifs (ITSMs) and the subsequent interaction of the SLAM-associated protein (SAP) adapter and other signaling molecules [10,11,12]. This initiates a series of signaling events that leads to the generation of the immunological synapse and the directed release of cytolytic granules containing perforin and granzymes by T lymphocytes and NK cells. Both 2B4 and CD48 contain an extracellular region composed of an N-terminal V-type Ig-like domain followed by a C-type Ig-like domain. CD48 binds with high affinity to 2B4, an interaction that occurs via their N-terminal domains and that is conserved throughout evolution among species. In addition, CD2 has been identified as a low-affinity receptor for CD48 [13].

Cytomegaloviruses (CMVs) are highly prevalent beta-herpesviruses that infect a wide range of species, including small mammals, humans, and other primates [14]. Reflecting their extensive co-evolution with their hosts, CMVs have adapted to be highly species specific. After primary infection, these viruses are not completely cleared but rather they establish latency and persist in their hosts for life, getting reactivated when their immune system is compromised. To achieve this persistence, CMVs have developed a remarkable variety of strategies to subvert immune surveillance [15,16]. Indeed, within their long double-stranded DNA genomes, which have the capacity to encode hundreds of gene products, these pathogens express a significant number of immunoevasins, proteins aiming to disrupt primary immune effector mechanisms [17]. Of particular importance are those directed to counteract NK and T cell functions. Interestingly, some of these immunoevasins derive from host genes, captured by CMVs via lateral gene transfer during their evolutionary history. Thus, CMVs typically bear homologs of MHC class I molecules, Fc receptors, cytokines, chemokines, and chemokine receptors [18,19,20].

We previously reported the presence of several CD48 homologs (vCD48s) encoded by cytomegaloviruses and other large DNA viruses [21,22]. In particular, two primate CMVs, owl monkey CMV (OMCMV) and squirrel monkey CMV (SMCMV), encode several copies of vCD48s, derived from a common host CD48 ancestor gene acquired by retrotranscription [22]. In a recent study, we investigated A43 in detail, the member of this vCD48 gene family that exhibits the highest amino acid identity with its cellular counterpart, demonstrating that it serves as a soluble viral CD48 decoy receptor [23]. This soluble viral protein binds with high affinity and stability to 2B4, severely impairing the establishment of the immunological synapse between NK cells and CD48-expressing target cells, consequently blocking NK-cell cytotoxicity and IFN-γ production. So far, and despite their potential in modulating host immune responses, the properties and the biological significance of the other members of this vCD48 family remain elusive.

Here, we have analyzed the other four vCD48s, A44 and A45 encoded by OMCMV and S30 and S31 encoded by SMCMV. We find that they are highly divergent molecules and that, in contrast to A43, they do not recognize the host 2B4 receptor, suggesting that they have evolved to acquire alternative binding specificities. Importantly, we demonstrate that S30 is a soluble protein able to interact with the co-stimulatory molecule CD2, while S31 is a cell surface protein that binds a different unknown ligand expressed in leukocytes. Thus, our findings unveil a new class of viral immunoevasins based on the CD48 immune receptor.

## 2. Materials and Methods

### 2.1. Cell Culture and Viral Infections

The cell lines HEL299 (human embryonic lung fibroblast), COS-7 (green monkey fibroblast), and HEK-293T (human embryonic kidney) were obtained from the American Type Culture Collection (ATCC), and the owl monkey kidney cell line OMK (637-69) was from Sigma-Aldrich. HEL299, COS-7, and HEK-293T cells were cultured in Dulbecco’s modified Eagle’s medium, and OMK cells in alpha minimum essential medium, both supplemented with 2 mM glutamine, 1 mM sodium pyruvate, 50 U of penicillin per mL, 50 g of streptomycin per mL, and 10% fetal bovine serum. B lymphocytes were obtained from fresh blood samples of *Saimiri boliviensis* squirrel monkeys (Faunia, Madrid, Spain), and immortalized by infection with Epstein–Barr virus (EBV; provided by M. Plana (Institut d’Investigacions Biomèdiques August Pi i Sunyer, Barcelona, Spain)), as described previously for *Aotus trivirgatus* owl monkey B lymphocytes [23]. The resulting polyclonal B lymphocyte cell lines were cultured in RPMI-1640 medium, supplemented as indicated above. SMCMV was obtained from the ATCC (VR-1398) and OMCMV was kindly provided by A. Davison (Medical Research Council–University of Glasgow Center Virus Research, Glasgow, UK; [24]). Viral stocks were prepared by infecting at a low moi HEL299 cells with SMCMV and OMK cells with OMCMV. Cell supernatants were recovered when maximum cytopathic effect was reached, and then cleared of cellular debris by centrifugation at 1700× *g* for 10 min. Viral titers were determined by standard plaque assays on HEL299 or OMK cells. Except for viral stock preparations, infections included a centrifugal enhancement of infectivity step [25].

### 2.2. Plasmid Constructions

HA-A44, HA-A45, HA-S30, and HA-S31, expressing the full-length viral proteins without their corresponding signal peptides and with the hemagglutinin (HA) epitope at their N-terminal ends, were generated as follows: first, DNA sequences were PCR-amplified using template DNA extracted from OMCMV or SMCMV particles and primer sets with restriction sites at the 5′ and 3′ ends. The resulting PCR products were inserted into the pGEM-T vector (Promega, Madison, WI, USA), subsequently digested, and cloned into the mammalian expression vector pDisplay (Merck Millipore, Burlington, MA, USA). A44-Fc, A45-Fc, S30-Fc, and S31-Fc fusion proteins, expressing the two Ig domains of these molecules (with the CD33 leader peptide replacing their own signal peptide) fused to the Fc region of human IgG1, were obtained by PCR using as templates the respective HA-vCD48 constructs, and specific sets of primers with restriction sites. The PCR-amplified products were inserted into pGEM-T and finally cloned into the pCI-neo Fc vector, as described before [22]. HA-sbCD2-Tm contains the ectodomain of *Saimiri boliviensis* CD2 (GenBank accession number XM_003933520.1) with an HA-tag at the N-terminal fused to the platelet-derived growth factor receptor (PDGFR) transmembrane domain (Tm). The DNA sequence of sbCD2 without its signal peptide was chemically synthesized (Genscript, Piscataway, NJ, USA) and cloned in frame with the HA-tag at the N-terminal and the PDGRF Tm at the C-terminal into the pDisplay vector, using restriction sites added at the 5′ and 3′ ends. CD58-Fc and HA-atCD2-Tm, containing the *Aotus trivirgatus* CD58 ectodomain fused to the Fc region of human IgG1 or the N-terminal HA-tagged ectodomain of *Aotus trivirgatus* CD2 fused to the PDGFR Tm domain, respectively, were constructed as follows: first, PCR products were generated using template DNA extracted from OMK cells and primer sets based on regions flanking the second and third exons, corresponding to the first and second Ig domains, respectively, that were conserved in *Aotus nancymaae*, *Callihtrix jacchus*, and *Saimiri boliviensis* CD58s and CD2s. The GenBank transcript annotations of the CD2 sequences were XM_012458868.1, NM_001257217.1, and XM_003933520.1, and for the CD58 sequences they were XM_012458872.1, XM_009001961.1, and XM_010344065.1, respectively. The resulting PCR products were inserted into the pGEM-T vector (Promega) and sequenced. The newly identified nucleotide sequences were deposited in GenBank under the following accession numbers: MT512625 for exon2 and exon3 of *Aotus trivirgatus* CD58, and MT512624 for exon2 and exon3 of *Aotus trivirgatus* CD2. Splicing by overlap extension (SOE) PCR was then performed to join sequences coding for the first and second Ig domains of these molecules. PCR amplifications were carried out employing the pGEM-T plasmids generated as templates, two internal sequence-complementary primers annealing with the final of the first and the beginning of the second Ig domains, and two external primers (with restriction sites at 5′ and 3′ ends). The resulting PCR products were annealed and subsequently cloned in the pCineo Fc vector in the case of CD58 and in the pDisplay plasmid in the case of CD2. HA-A43, A43-Fc, *Aotus trivirgatus* HA-2B4 (HA-at2B4), and *Aotus trivirgatus* CD48-Fc were previously described [22,23]. The pDisplay HA-sb2B4, expressing the N-terminal HA-tagged ectodomain of *Saimiri boliviensis* 2B4 (GenBank accession number XM_003937960.2) fused to a region of human 2B4 (from residue 219 in the stalk segment to stop codon), was constructed by PCR following the same indications as for HA-at2B4 [23] and using sb2B4 without its signal peptide chemically synthesized (Genscript) as template. All PCR reactions were performed under the following conditions: one cycle at 94 °C for 5 min; 30 cycles of 1 min at 94 °C, 1 min at 51 °C, and 1 min at 72 °C; and one cycle at 72 °C for 10 min. For the annealing reactions, the conditions were: six cycles of 5 min at 94 °C; one cycle at 51 °C for 1 min; one cycle at 72 °C for 1 min; and one cycle of 10 min at 72 °C. Primers used for the construction of the different plasmids are shown in Table 1. The identification of all recombinant plasmids was confirmed by DNA sequencing.

### 2.3. Reverse Transcriptase PCR

OMK or HEL299 cells were mock infected or infected with OMCMV or SMCMV, respectively, at an moi of 1. The chemical inhibitors cycloheximide (CHX; 100 µg/mL; Sigma-Aldrich, St. Louis, MO, USA) or phosphonoacetic acid (PPA; 250 µg/mL; Sigma-Aldrich) were used to assess the selective expression of viral immediate early genes or early genes, respectively. Cultures were treated with CHX 30 min before infection, or with PPA at the time of infection, and both inhibitors were maintained until RNA was harvested. Total RNA was isolated at different times after infection (13 h post infection for CHX samples and 72 h post infection for the rest) by using the TRIzol method (Invitrogen, Thermo Fisher Scientific, Waltham, MA, USA). Reverse transcriptase-mediated PCR (RT-PCR) was then performed employing the SuperScript III First-strand Synthesis System for RT-PCR (Invitrogen) according to the manufacturer’s protocol. Control reactions were carried out in the absence of RT to analyze the specific detection of RNA. Amplified products (a 588-bp fragment for A44; a 637-bp fragment for A45; a 590-bp fragment for OMCMV IE1; a 570-bp fragment for OMCMV UL54; a 166-bp fragment for OMCMV UL73; a 598-bp fragment for S30; a 638-bp fragment for S31; a 360-bp fragment for SMCMV IE1; a 570-bp fragment for SMCMV UL54; a 167-bp fragment for SMCMV UL73; and a 101-bp fragment for GAPDH) were separated on a 1% agarose gel and visualized by RedSafeTM nucleic acid staining solution (iNtRON Biotechnology Inc., Gyeonggi-do, Korea).

### 2.4. Transfections, Generation and Quantification of Fc Fusion Proteins

COS-7 cells were transiently transfected with 5 µg of the indicated plasmid using the Amaxa Cell Line Nucleofector Kit R according to the manufacturer’s protocol. To generate soluble Fc fusion proteins of each vCD48, CD48, and CD58, HEK-293T cells were transiently transfected with 0.2 µg/cm^2^ of the indicated plasmid mixed with 6 µL/µg DNA of polyethylenimine (1 mg/mL, Sigma-Aldrich) in 0.1 mL/cm^2^ of OPTIMEM medium (Gibco, Thermo Fisher Scientific) for four hours. Then, cultures were washed and 6 days later the supernatants containing the Fc fusion proteins were collected, clarified to remove cellular debris, and concentrated 20-fold using the Amicon Ultra-15 Centrifugal Filter Unit with an Ultracel-30 membrane (Merck Millipore). The quantification of Fc fusion proteins was performed by sandwich ELISA employing anti-human Fc IgG mAb (clone 29.5; Fc specific; [22]) and anti-human IgG (Fc specific; Sigma-Aldrich) peroxidase (POD).

### 2.5. Flow Cytometry Analysis

Flow cytometry was performed using standard procedures [26]. To determine the cell surface expression of HA-tagged proteins, COS-7 cells were stained with the anti-HA mAb [22], followed by anti-mouse IgG-PE (Jackson ImmunoResearch, Ely, Cambridgeshire, UK). For Fc fusion protein staining, 8 µg/mL of each Fc fusion protein were used, followed by incubation with the anti-human Fc IgG mAb and by anti-mouse IgG-PE. An irrelevant Fc fusion protein (CTL-Fc) was always used as a negative control. To minimize non-specific staining, all incubations were carried out in the presence of 20% rabbit serum (Linus) and 1% fetal bovine serum in PBS. Samples were analyzed using FACSCalibur (BD Biosciences, San Jose, CA, USA) and FlowJo software (Tree star Inc, Ashland, OR, USA).

### 2.6. Immunoprecipitations, Glycosidase Treatments, and Western Blot Analyses

Immunoprecipitations were performed on COS-7 cells non-transfected or transfected with HA-A45 or HA-S31, surface-labeled with biotin (Sigma-Aldrich) and lysed, or on concentrated supernatants of COS-7 cells non-transfected or transfected with HA-A44 or HA-S30. Samples were precleared three times for 30 min using protein G Sepharose (GE Healthcare, Chicago, IL, USA) and immunoprecipitated by incubation with anti-HA-agarose conjugate (Sigma-Aldrich). Immunoprecipitates were washed and eluted. Samples from total cell extracts were lysed and quantified by a BCA Protein Assay Kit (Thermo Fisher Scientific). When indicated, immunoprecipitates or lysed samples were treated with the *N*-glycosidase F deglycosylation kit or/and an *O*-glycosidase and neuraminidase bundle (New England BioLabs, Ipswich, MA, USA) following the manufacturer’s instructions. Samples from untreated or treated immunoprecipitates or total cell extracts were subjected to SDS-PAGE in 10% acrylamide gels and subsequently transferred to nitrocellulose membranes (Protran, Merck Millipore). Membranes were incubated with streptavidin-POD conjugate (Roche, Basel, Switzerland) when analyzing HA-A45 or HA-S31 immunoprecipitates, or rabbit anti-HA mAb (clone c2974; Cell Signaling MP) followed by anti-rabbit IgG-POD (Promega) when examining the rest of the samples. As a loading control, an anti-actin mAb (clone C4; MP Biomedicals, Irvine, CA, USA) was employed, followed by anti-mouse IgG-POD (Sigma-Aldrich). Blots were developed using a SuperSignal^®^ West Pico Chemiluminescent Substrate (Pierce, Thermo Fischer Scientific) according to the manufacturer’s protocol.

### 2.7. Sequence Analysis, Protein Domain and Motif Prediction, and Structure Modeling

Protein and nucleotide sequence alignments were obtained using MAFFT version 7.467 [27]. To calculate the percentage of amino acid identity, the sequences of each viral CD48 homolog and those of the *Aotus trivirgatus* or the *Saimiri boliviensis* CD48 protein were paired and aligned and positions containing gaps were discarded. The initial calculation of pairwise amino acid identity and similarity (positives) of the vCD48 proteins was obtained using BLAST-Global Align [28], adjusting the values after removing the positions with gaps. Ig domains were determined from annotations in Conserved Domain Database (CDD) [29]. Signal peptides and transmembrane regions were predicted by using SignalP 4.1 [30] and TMHMM 2.0 [31], respectively. Protein *N*-glycosylation and *O*-glycosylation sites were identified by using NetNGlyc 1.0 [32] and NetOGlyc 4.0 [33], respectively. Structure modeling of the viral and host CD48 proteins was performed using SWISS-MODEL with the template 2ptt, which concerns the mouse CD48 structure [34,35].

## 3. Results

### 3.1. Genomic and Protein Diversity of the VCD48 Gene Family

**Figure 1:**
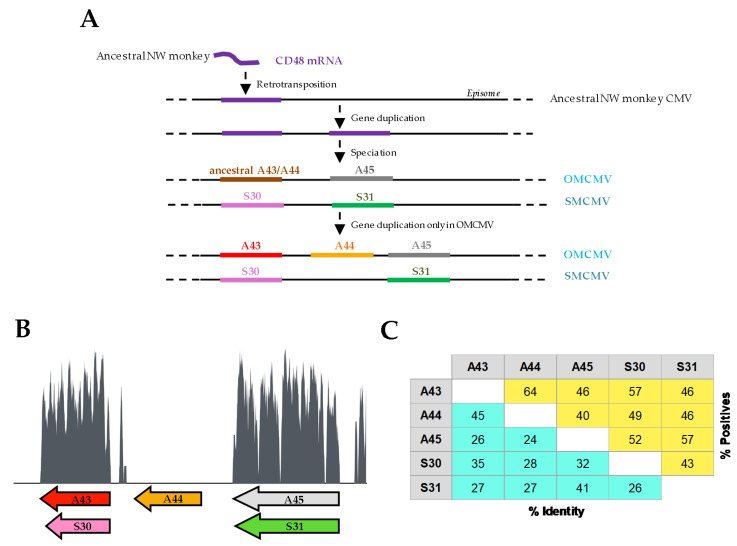
Evolution and diversity of the vCD48 family. (**A**) Schematic representation of the predicted evolution of the vCD48 gene family. (**B**) Sliding window plot of the nucleotide conservation along the vCD48 genomic region between owl monkey cytomegalovirus (OMCMV) and squirrel monkey CMV (SMCMV). Bottom arrows annotate the location of the members of this gene family. A sliding window of 30 nt was used to calculate the plot from a nucleotide alignment obtained using MAFFT. (**C**) Percentage of amino acid identity and similarity (positives) between the vCD48 proteins encoded by these CMVs. These percentages were calculated for each pair of amino acid sequences using BLAST (Global Align option) adjusting the values after removing the positions with gaps.

The vCD48 family members, A43, A44, and A45 in OMCMV, and S30 and S31 in SMCMV, are arranged in tandem toward the end of the corresponding CMV genomes within the unique short (US) region. Our previous phylogenetic analysis indicated that the origin of these vCD48 genes was a unique event of gene capture from the genome of a New World (NW) monkey ancestor of *Aotus trivirgatus* and *Saimiri boliviensis*, the hosts of OMCMV and SMCMV, respectively [22]. This event was followed by a duplication of the captured gene and the posterior speciation of these two CMVs (Figure 1A). An additional gene duplication episode took place only in OMCMV, resulting in three vCD48s, A43, A44, and A45, whereas SMCMV encodes only two, S30 and S31. To further investigate how these vCD48 genes and their encoding proteins have diverged, we analyzed the nucleotide conservation along the genomic region where these genes are located and the degree of amino acidic divergence between each pair of vCD48s. To this end, we generated a pairwise alignment of OMCMV and SMCMV genomic sequences covering the vCD48 region and plotted the degree of nucleotide conservation along these positions (Figure 1B). The two fragments that show the highest conservation correspond to the A43–S30 and A45–S31 pairs, whereas no conservation is observed for A44, adding support to our proposed model for the vCD48 family evolution (Figure 1A). In addition, we calculated the percentage of amino acid identity of the vCD48 proteins, in pairs, using BLAST with the Global Align option (Figure 1C; [28]). This tool also computes the percentage of positives, an estimation of amino acid similarity. It is interesting to note that the pair A45–S31 (split as result of the speciation event) shows a level of amino acid conservation similar to the pair A43–A44 (the product of a more recent event, a gene duplication in OMCMV) with percentages of identity/positives of 41%/57% and 45%/64%, respectively. This result points to the probable maintenance between A45 and S31 not only of the protein structure but also their function. Moreover, it is particularly interesting to observe that, whereas the percentage of positives is the same for A43–S30 and A45–S31 (57%), the percentage of identity is clearly lower for A43–S30 (35% comparing with 41%). This suggests constraints on the evolution of S30 to maintain the structure of the protein, as indicated by the percentage of positives, but important amino acid changes that could alter its function.

### 3.2. Determination of the Kinetic Class of the VCD48 Family Members

**Figure 2:**
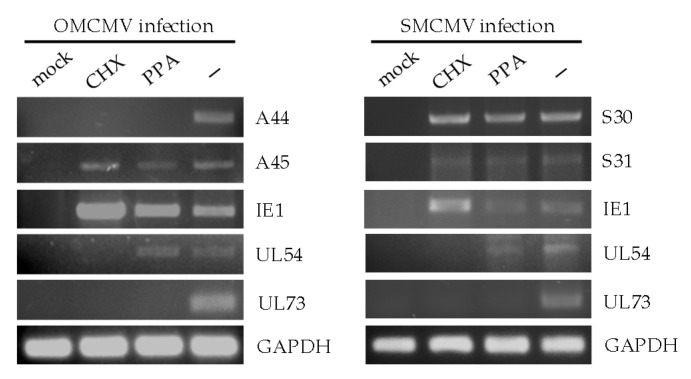
Expression of *vCD48s* during OMCMV and SMCMV infections. Owl monkey kidney (OMK) cells (left panels) or HEL299 (right panels) cells were mock infected or infected with OMCMV or SMCMV at an moi of 1 in the absence (-) or presence of cycloheximide (CHX) or phosphonoacetic acid (PPA), as indicated in the Materials and Methods. Whole-cell RNAs were harvested at 13 hpi in the case of CHX samples and at 72 hpi for the rest of the samples, and subsequently reverse transcribed. PCRs were performed using primer sets specific for OMCMV genes *A44*, *A45*, *IE1*, *UL54*, and *UL73*, SMCMV genes *S30*, *S31*, *IE1*, *UL54*, and *UL73*, or *GAPDH*, as shown. Amplified products were separated on 1% agarose gels and visualized by RedSafe nucleic acid staining solution.

Like in all herpesviruses, CMV gene expression during the lytic cycle takes place in a tightly regulated sequential manner, and viral genes can be classified into three major kinetic groups, termed immediate early (IE), early, and late. To gain a first insight into the vCD48s that remained unexplored, A44, A45, S30, and S31, we analyzed their expression during productive infection and determined their kinetic class. Thus, owl monkey kidney (OMK) epithelial cells or human embryonic HEL 299 cells were mock infected or infected at an moi of 1 with OMCMV or SMCMV, respectively, in the absence or presence of two classical chemical inhibitors: CHX, which blocks protein synthesis, or PPA, which prevents viral DNA replication. Total RNA was extracted from the cultures and RT-PCR analysis was performed using specific primers for each vCD48. As shown in Figure 2, A45, S30, and S31 were detected under all conditions tested, including in the presence of CHX, following an expression pattern similar to that of the immediate early OMCMV gene IE1. In contrast, A44 was found to be sensitive not only to the treatment with CHX, but in addition to PPA, a condition in which, as expected, the early viral polymerase was expressed. Thus, A44 was only detected in the absence of inhibitors, as it occurs with the late UL73 virion envelope N gene. These results indicate that, while vCD48s A45, S30, and S31, as we previously reported for A43 [23], are immediate early genes, A44 can be classified as a late gene. The findings also suggest that the vCD48s of OMCMV are controlled by distinct promoters.

### 3.3. Biochemical Characterization and Cellular Localization of A44, A45, S30, and S31

**Figure 3:**
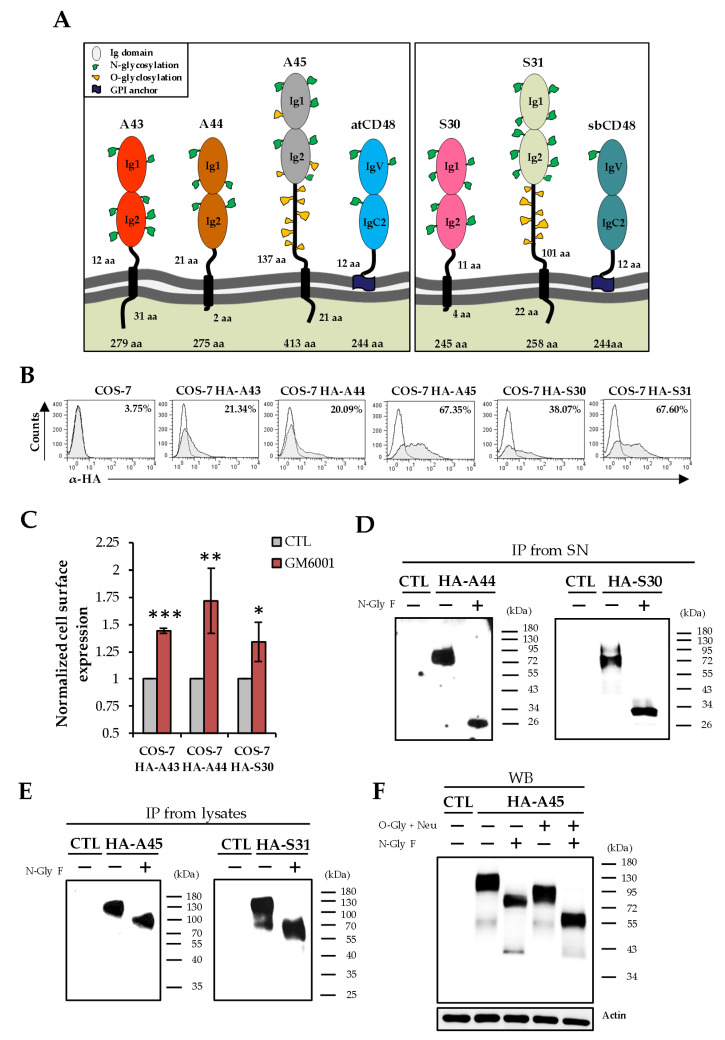
Expression and cellular localization of the vCD48 proteins. (**A**) Graphical illustration of the A43, A44, A45, S30, and S31 proteins and their cellular counterpart CD48s. The lengths of each of the proteins, their membrane proximal regions, and their cytoplasmic tails are indicated. For clarity, in A45 and S31, only a number of the predicted O-glycosylations are shown. (**B**) Flow cytometry analysis of COS-7 cells non-transfected or transfected with HA-A43, HA-A44, HA-A45, HA-S30, or HA-S31 and stained with anti-hemagglutinin (HA) monoclonal antibody (α-HA, shaded histograms) or an isotype control (open histograms). The percentage of positive cells is indicated in each histogram. Results are representative of at least three independent experiments. (**C**) Analysis by flow cytometry of the normalized cell surface expression of HA-A43, HA-A44, or HA-S30 transfected in COS-7 cells and untreated (CTL) or treated with 20 µM of the metalloproteinase inhibitor GM6001 for 24 h. Means ± standard deviations of three independent experiments are shown. A two-tailed Student’s *t*-test was used to assess statistical differences between experimental groups. * *p* < 0.05, ** *p* < 0.01, *** *p* < 0.001. (**D**) The supernatants (SN) of COS-7 cells non-transfected (CTL) or transfected with HA-A44 or HA-30 were immunoprecipitated (IP) with an anti-HA mAb. (**E**) COS-7 cells non-transfected (CTL) or transfected with HA-A45 or HA-S31 were surface labeled with biotin, lysed, and then immunoprecipitated (IP) with an anti-HA mAb. Where indicated, cell lysates were treated with N-glycosidase F (N-Gly F). (**F**) COS-7 cells non-transfected (CTL) or transfected with HA-A45 were lysed and, where indicated, treated with N-glycosidase F (N-Gly F), O-glycosidase, and neuraminidase (O-Gly + Neu), or a combination of them (N-Gly F, O-Gly + Neu). In **D**–**F**, samples were subjected to SDS-PAGE and subsequent western blot analysis using an anti-HA mAb followed by anti-rabbit IgG-peroxidase (POD; **D**,**F**), or streptavidin-POD (**E**). An anti-β-actin mAb followed by anti-mouse IgG-HRP was used as an internal control in F. Molecular masses (in kilodaltons) are indicated.

We then sought to explore the properties of these viral proteins. Unlike CD48, vCD48s are predicted to be type I transmembrane proteins. They are composed of two extracellular Ig-like domains and distinctive proximal transmembrane regions and cytoplasmic tails (Figure 3A). In addition, a notable feature of A45 and S31 is their long stalks. To analyze the expression of these viral proteins, we constructed plasmids encoding N-terminal HA-tagged versions of each of them (named HA-A44, HA-A45, HA-S30, and HA-S31). COS-7 cells were transiently transfected with these plasmids and examined by flow cytometry using an anti-HA-specific antibody. As shown in Figure 3B, A45 and S31 were abundantly expressed at the cell surface. In contrast, A44 and S30 were found to be minimally detected at this cellular location, suggesting that these proteins, as in the case of A43 [22,23], might be cleaved from the plasma membrane and their ectodomains shed to the extracellular space. Consistent with this notion, when HA-A44, HA-S30, or HA-A43 transfected cells were treated with GM6001 (Merck Millipore), a broad-spectrum inhibitor of zinc-dependent metalloproteases, a substantially increased surface staining was observed for the three proteins (Figure 3C). In addition, when we examined the extracellular media from COS-7 cells transfected with HA-A44 or HA-S30 by immunoprecipitation using anti-HA agarose beads followed by western blot analysis, the A44 and S30 proteins were detected as a 76–97 kDa or a 65–93 kDa band, respectively (Figure 3D). Taken together, these results indicate that both A44 and S30 are released from the cell through their proteolytic processing.

To determine the molecular mass of A45 and S31 and further corroborate the presence of these viral proteins at the cell membrane, HA-A45 or HA-S31 transfected COS-7 cells were surface labeled with biotin, immunoprecipitated with anti-HA agarose beds and analyzed by western blot using, in this case, labeled streptavidin. As shown in Figure 3E, A45 was visualized as a 105–137 kDa band and S31 as a 95–131 kDa band. Taking into account the molecular weights observed for the four vCD48s analyzed, together with the prediction of multiple potential *N*-glycosylation sites in their ectodomains (five in A44, six in A45, seven in S30, and 10 in S31, Figure 3A) we next examined their glycosylation extent. Samples from HA-immunoprecipitated cell lysates (for HA-45 and HA-S31) or from the extracellular medium (for HA-44 and HA-30) of transfected cells were treated with *N*-glycosidase F enzyme and subsequently examined by western blot. Accordingly, a marked increase in the electrophoretic mobility of the four proteins upon *N*-glycosidase treatment was obtained (Figure 3D,E), evidencing their high levels of N-glycosylation. A45 and S31 were also predicted to be heavily O-glycosylated (57 potential sites in A45 and 28 in S31; Figure 3A). Taking A45 as an example of these two proteins, we analyzed its O-glycosylation extent. When lysates of HA-A45 transfected COS-7 cells were digested with *O*-glycosidase and neuraminidase, A45 migrated with a substantially reduced size (Figure 3F). Moreover, combined treatment of *N*-glycosidase F, *O*-glycosidase, and neuraminidase led to an additional shift of the major A45 band, results that confirmed the presence of O-linked glycans in this viral protein. Thus, altogether, these data indicate that the vCD48s are very different molecules, diverging in some important attributes, such as their cellular location, biochemical composition, and temporal expression kinetics during infection.

### 3.4. Analysis of the Capacity of VCD48S to Bind to Host 2B4

**Figure 4:**
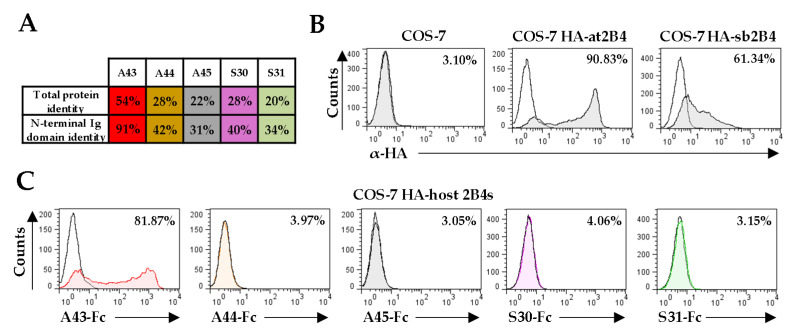
Analysis of the binding capacity of A44, A45, S30, and S31 to host 2B4. (**A**) Amino acid identity between each vCD48 and host CD48. The percent amino acid identity for the complete sequence or the N-terminal Ig domain sequence of each vCD48 compared to those of the corresponding host CD48 is shown. (**B**) Flow cytometry analysis of COS-7 cells non-transfected or transfected with HA-at2B4 or HA-sb2B4 and stained with α-HA mAb (shaded histograms) or an isotype control (open histograms). The percentage of positive cells is indicated in each histogram. (**C**) Interactions of vCD48s with host 2B4. COS-7 cells transfected with each host 2B4 as indicated in B were incubated with 8 µg/mL of A43-Fc, A44-Fc, or A45-Fc in the case of HA-at2B4 transfected cells or S30-Fc and S31-Fc in the case of HA-sb2B4 transfected cells and then analyzed by flow cytometry (shaded and colored histograms). Open histograms represent isotype controls. The percentage of positive cells is indicated in each histogram.

The sequence identity shared between the N-terminal Ig domain of host CD48s and those of A44, A45, S30, and S31 are relatively low (between 31–42%; Figure 4A). However, as it occurs with several other CMV homologs of cellular proteins that have retained the functions of the original molecule despite having diverged considerably, it seemed feasible that the viral CD48 homologs would bind host 2B4 [18,21]. Thus, to explore this aspect, we constructed soluble vCD48-Fc fusion proteins, comprising the extracellular region of each viral protein fused to the Fc domain of the human IgG1, and assessed their capacity to interact with COS-7 cells transiently transfected with plasmids that expressed HA versions of either host 2B4 (HA-at2B4 or HA-sb2B4) at the cell surface (Figure 4B). As illustrated in Figure 4C, only the A43-Fc protein efficiently recognized at2B4, while none of the other four viral proteins, A44-Fc, A45-Fc, S30-Fc, or S31-Fc, was able to bind to their corresponding host 2B4. These results indicate that viral CD48s have differentially evolved in the viral genome, with only A43 retaining CD48 ligand abilities.

### 3.5. Predicted Tertiary Structure of the VCD48 N-Terminal Ig Domains

**Figure 5:**
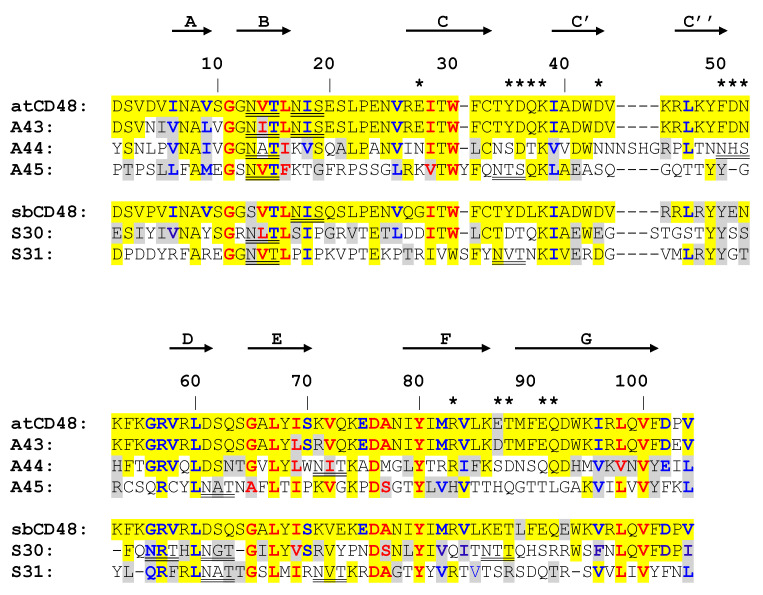
Alignment of the N-terminal Ig domains of vCD48s and host CD48s. The predicted β-strands are depicted with arrows at the top and labeled using the standardized nomenclature for Ig-like domains: A, B, C, C’, C’’, D, E, F, and G. Potential N-linked glycosylation sites are double underlined. Conserved residues (with respect to CD48) are shaded in yellow (identical amino acid) or light gray (similar amino acid). Amino acids colored in bold red are conserved between most members of the Ig superfamily; those colored in bold blue are conserved between most SLAM family receptors. Contacting residues in the 2B4:CD48 heterophilic dimer are marked using black stars above the atCD48 sequence.

To try to understand the molecular basis of the differential interaction of A43, A44, A45, S30, and S31 with host 2B4, we examined in more detail the sequence and structure of their N-terminal Ig domains. To this end, the sequence of the IgV-like domain of each host CD48 and those of the viral homologs were aligned. As illustrated in Figure 5, the five vCD48s conserved nearly all residues common to most Ig superfamily members (red residues), as well as those characteristic of SLAM family receptors (blue residues). However, when we inspected residues involved in the CD48:2B4 interaction (residues marked with stars; [34]), around 70–80% of them have been substituted for very different amino acids in A44, A45, S30, and S31 (Figure 5). In contrast, only one of these 14 amino acids has changed in A43 (E87 to D87).

**Figure 6:**
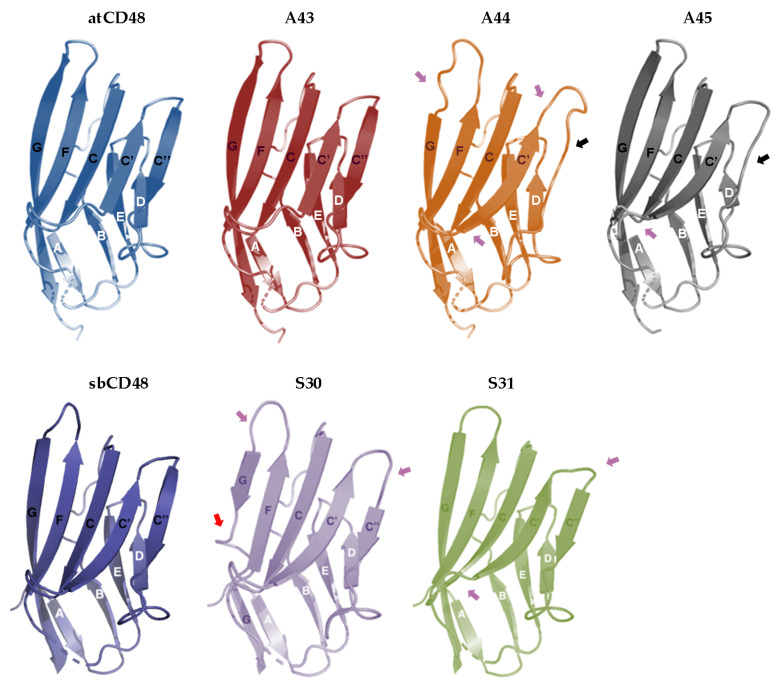
Predicted tertiary structure of host CD48s and vCD48s. β-strands are shown as arrows labeled as in Figure 5. The absence of the predicted C’’ β-strands in A44 and A45 is indicated by black arrows, the truncated G β-strand in S30 by a red arrow, and the different CC’, C’C’’ or C’D, and FG loops in the vCD48s as compared to host CD48s by pink arrows.

We also molecularly modeled these domains based on the crystal structure of the murine CD48 receptor (Figure 6; [34]). The IgV-like N-terminal domain of each host CD48 contains nine β-strands assembled into two antiparallel β-sheets designated AGFCC’C’’ and DEB, with the AGFCC’C’’ β-sheet interacting with 2B4 (Figure 5 and Figure 6). The analysis revealed that the five viral proteins preserved the characteristic Ig superfamily β-sandwich fold of this domain with an overall similar topology. However, we found that A44 and A45 differ from host CD48 in the number (eight instead of nine) and length of their β-strands. These two viral proteins lack the predicted C” β-strand, and the C’ β-strand is slightly longer than that of CD48 (Figure 5 and Figure 6). In addition, a shorter G β-strand is predicted for the A44 IgV-like domain. Concerning S30 and S31, their N-terminal domains are composed of the same number of β-strands than host CD48, but with significant length differences. For example, S30 contains a truncated G β-strand, and in both, S30 and S31, the predicted C” β-strand is shorter than that of host CD48. Importantly, the indicated differences in the β-strands of the four vCD48s in turn impact on the length of the neighboring inter-sheet loops, which are key to the interaction with 2B4. Finally, while the predicted N-glycosylation sites in the IgV-like domain of host CD48s and A43 lie outside the binding interface, A44, A45, S30, and S31 contain additional potential linked glycosylation sites, one of them in each protein placed in positions within this interface of 2B4 interaction (Figure 5). Thus, altogether, these observations may account for the lack of recognition of host 2B4s by A44, A45, S30, and S31. In contrast, the A43 folding was indistinguishable from that of CD48, consistent with this viral protein being a very close mimic of the host immune receptor and capable of interaction with 2B4.

### 3.6. Analysis of the Ability of vCD48s to Recognize Host CD2

**Figure 7:**
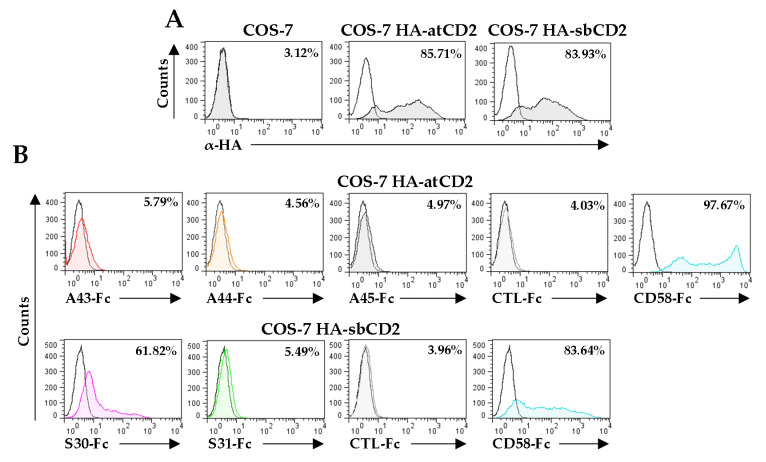
Analysis of the binding capacity of A44, A45, S30, and S31 to host CD2. (**A**) Flow cytometry analysis of COS-7 cells non-transfected or transfected with HA-atCD2 or HA-sbCD2 and stained with α-HA mAb (shaded histograms) or an isotype control (open histograms). The percentage of positive cells is indicated in each histogram. (**B**) Interactions of vCD48s with host CD2. COS-7 cells transfected with either HA-atCD2 (upper panels) or HA-sbCD2 (bottom panels) were incubated with 8 µg/mL of A43-Fc, A44-Fc, A45-Fc, S30-Fc, S31-Fc, atCD58-Fc, or an unrelated CTL-Fc fusion protein, as indicated, and then analyzed by flow cytometry (shaded and colored histograms). Open histograms represent isotype controls. The percentage of positive cells is indicated in each histogram.

The fact that these vCD48s do not bind 2B4 but preserve the overall Ig structure of their N-terminal domains likely suggests that they have diverged to interact with alternative immune receptors. CD2 is a cell surface protein expressed by T lymphocytes and NK cells that binds to its ligand CD58 on antigen-presenting cells in humans, playing an important role in strengthening adhesion to promote T cell activation. In addition, human CD2 can also interact with a very low affinity to CD48 (dissociation constant <0.5 mM). Indeed, in rodents that lack CD58, CD48 is the ligand of CD2 [13]. This prompted us to explore whether A44, A45, S30, and S31 could interact with host CD2. To assess it, COS-7 cells transiently transfected with either HA-atCD2-Tm or HA-sbCD2-Tm, plasmids that express the ectodomain of CD2 from *Aotus trivirgatus* and *Saimiri boliviensis*, respectively (Figure 7A), were tested in binding assays performed, by cytometry, with each of the vCD48-Fc fusion proteins. In addition, a host CD58-Fc fusion protein and an unrelated fusion protein were included as controls of the assay. Figure 7B shows that, while no interaction of A43-Fc, A44-Fc, A45-Fc, or S31-Fc with transfected cells could be observed, S30 efficiently recognized sbCD2 (Figure 7B). Thus, the viral S30 protein presents shifted binding properties, as it is able to interact with host CD2, but no longer with host 2B4.

**Figure 8:**
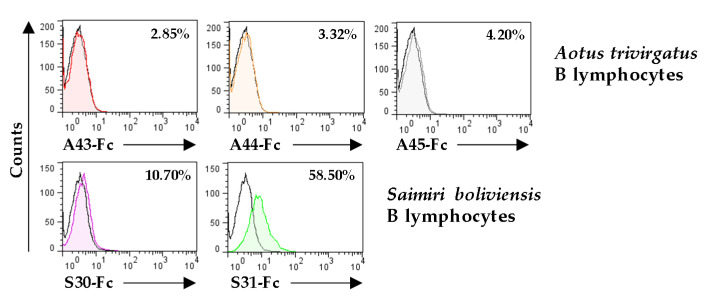
Analysis of the binding capacity of vCD48s to Epstein–Barr virus (EBV)-immortalized B lymphocytes. EBV-immortalized *Aotus trivirgatus* (top panels) or *Saimiri boliviensis* (bottom panels) B lymphocytes were incubated with 8 µg/mL of each of the indicated vCD48-Fc fusion protein and analyzed by flow cytometry (shaded and colored histograms). Open histograms represent isotype controls. The percentage of positive cells is indicated in each histogram.

Due to the difficulties in obtaining cytotoxic leukocytes from these two New World monkeys that could allow us to further identify potential targets of the remaining ligand orphan vCD48s, the A44, S31, and A45 molecules, we decided to immortalize B lymphocytes with EBV and used them to test whether they were recognized by any of these viral proteins. As illustrated in Figure 8, when flow cytometry analyses were performed employing the vCD48-Fc fusion proteins, we could determine that one of them, the S31 protein, was capable of binding to the B cell line. The fact that these cells do not express CD2 or 2B4 indicates that S31 has diverged to recognize a new, and still unknown, ligand.

## 4. Discussion

During millions of years of co-evolution, CMVs have acquired genetic material from their hosts through horizontal gene transfer. This process allows viruses to enhance their adaption to the environment and contributes to the renovation of their immune evasion strategies [36,37]. We previously described that two CMVs that infect New World monkeys, OMCMV and SMCMV, encode a number of SLAM receptor homologs, which include several copies of vCD48s [21,22,23]. Our interest was originally directed towards one of these vCD48s, the OMCMV A43 protein, and showed that it is a soluble CD48 decoy molecule, capable of binding with high affinity to 2B4 and protecting CD48-expressing cells against 2B4-mediated NK cell toxicity [23]. In this study, we have characterized the rest of the vCD48s, finding that they are disparate molecules, displaying unique traits and ligand-binding profiles.

Gene duplication plays a prevalent role in herpesvirus evolution, providing raw genetic material for innovation, and frequently leading to the emergence of new gene families, with their members often clustered together in the viral genome [14,38,39]. The v*CD48s*, *A43*, *A44*, *A45*, *S30*, and *S31* genes appear to have been derived from a common captured host CD48 ancestor by two gene duplication events, one before and the other after the split of the two monkey CMVs, and subsequent differentiation, constituting a family that is organized as a tandem head-to-tail gene array in the terminal region of the two viral genomes [22]. Interestingly, and in contrast to the host CD48 that is a GPI-anchored molecule, the five products of these genes have a skeleton of type I membrane proteins, with their extracellular regions, in all cases, preserving the two-Ig domain composition of their cellular counterpart. However, we found that these viral proteins differ markedly among themselves, not only in sequence and structure, but also in their biochemical properties, as well as in their spatial patterns and temporal kinetics during infection. Due to the lack of specific antibodies against these viral molecules, their features were mainly analyzed in transient transfection assays. In this connection, we must take into account that the properties of the CMV-encoded SLAM receptor homologs examined in detail to date, such as the OMCMV vCD48 A43 [23] or the human CMV (HCMV) vCD229s [40], when assessed in overexpression systems, closely correlate with those observed during infection. Hence, we determined that two of the vCD48s, A45 and S31, which are the ones displaying long stalks, are expressed at the cell surface, whereas A44 and S30, in a similar way to A43, are proteolytically processed and shed to the extracellular milieu [23]. This later feature is remarkable, as to date only a limited number of CMV proteins have been reported to be secreted, and among them, interestingly, we identified the homologs of the CD229 SLAM receptor present in HCMV and OMCMV [22,40,41,42]. Hyper-glycosylation, an important post-translational modification affecting the stability, immunogenicity, and function of viral proteins, has been frequently associated with CMV homologs of cellular proteins that participate in immune evasion [43,44]. Notably, we observed that the ectodomains of the vCD48s are heavily N-glycosylated, and in the case of A45 and S31, also extensively O-glycosylated, a process most likely intended to protect their stalk regions from proteolysis. Lastly, the expression of the vCD48 genes seems to be controlled by separate promoters, at least in OMCMV. Our data indicate that A44 is produced in the late phase of viral infection, while the rest of the vCD48s are synthesized with immediate early kinetics.

The observed diversification and refinement of these viral CD48 homologs throughout evolution suggest that they have been adapted to target different ligands. Consistent with this, when we inspected the N-terminal Ig domain of these proteins by aligning their sequences and modeling them based on the murine CD48 crystal structure [34], we observed that, while the A43 interaction interface closely mimics that of CD48, the corresponding sequences in A44, A45, S30, and S31 present notable modifications that explain their lack of recognition of 2B4. In addition, it must be noted that, as compared with host CD48s, these four vCD48s, but not A43, contain at least one additional potential N-glycosylation site within the predicted residues involved in ligand interaction. These results suggest that A44, A45, S30, and S31 no longer function as CD48 mimics and have been molded to perform 2B4-independent functions. Indeed, the classical model for the evolution of duplicate genes implies that one copy often evolves more slowly than the other and retains ancestral function, whereas the other copy is free to evolve more rapidly because it has no constraints, allowing it to therefore yield a gene with slightly modified or completely new functional properties [45]. Thus, considering that OMCMV counts with A43 that retain the CD48 original task, this would imply that A44 and A45 would have tolerated variations in their binding surfaces to accommodate new ligand interactions.

Importantly, we found that one of the vCD48s encoded by SMCMV, S30, can recognize a host crucial molecule, CD2, whose primary ligand in humans and primates is CD58. CD48 and CD2 are structurally related molecules. Indeed, CD2, CD58, and the SLAM receptors belong to the greater CD2 Ig superfamily, which groups its eleven members mainly based on homology within their extracellular domains [46]. Moreover, in some species, as mice or rats, that lack CD58, the ligand of CD2 is CD48 [47,48]. In humans and other primates, however, the CD2:CD48 interaction is of very low affinity and unlikely to be of functional significance [49]. It must be taken into account that the overall architecture of the 2B4:CD48 and CD2:CD58 complexes is roughly similar, with binding interfaces mainly formed by polar amino acids, although with some differences in the distribution of salt bridges and hydrogen bonds [34,50]. Thus, just several amino acid changes in the contact surface of the S30 protein could explain its switch of ligand from 2B4 to CD2, recovering a nearly extinct binding capacity. The human CD2 co-stimulatory molecule is expressed in T and NK cells and plays an important role in adaptive immunity, mediating adhesion and participating in the formation of the immune synapse between T lymphocytes and antigen-presenting cells. Moreover, CD2 is also critically involved in innate immunity, acting as a co-stimulating receptor between NK cells and target cells [51,52,53,54,55]. Owing to the overall S30 protein properties, and in particular, to its soluble nature, it is reasonable to think that this viral product could act as a CD58 viral decoy receptor during SMCMV infection, directly recognizing and blocking CD2 in T lymphocytes and NK cells, and consequently disrupting critical host processes, such as antigen presentation or NK cell-mediated cytotoxicity against infected cells. Remarkably, to our knowledge, this represents the first description of a soluble viral protein directly interacting with the cell surface co-stimulatory CD2 molecule.

Ig domains are particularly suitable for modulating recognition, adhesion, and communication processes between cells [56]. Thus, since the Ig fold of the N-terminal domain of the vCD48s is well preserved, one could anticipate that, as illustrated for A43 and S30, the A44, A45, and S31 proteins would also participate in this type of process, contributing to the mediation of viral immune evasion. Accordingly, we observed that the SMCMV S31 protein can interact with a molecule expressed on the surface of B lymphocytes. Due to the complications of obtaining host immune cells, examining the expression of the S31 binding partner in additional leukocyte subsets, and identifying its nature might not be a trivial task. Remarkably, and despite the nucleotide and amino acid conservation observed in our initial bioinformatics study between S31 and A45, we were unable to detect a similar binding of A45 to B lymphocytes, pointing to a functional divergence between both genes. In any case, the structural innovation of the long glycosylated stalks introduced in these two plasma cell surface proteins should be a critical determinant of their mechanisms of action. These viral proteins could be interrupting cell–cell communications by provoking a steric impediment that would distance the contact surface between the infected cell and the immune cell. In fact, it has been shown that an artificial increase in size of the ectodomains of CD2 and CD48 results in the substantial inhibition of antigen presentation [57]. On the other hand, it has also been reported that the presence of long stalks in Ig superfamily members could allow a better exposure of the Ig domains in the plasma membrane, favoring interactions with their ligands [58,59]. This might be of special relevance if these molecules work by modulating the activity of particular immune cells by engaging inhibitory receptors [60].

An intriguing question which arises from this study is why the loss of binding to 2B4 occurs in the two vCD48 members of SMCMV, in contrast to OMCMV, where A43 conserves the binding specificity to 2B4. In fact, one would predict that the most similar A43 homolog in SMCMV, S30, should have been the candidate vCD48 retaining this attribute. Interestingly, our preliminary BLAST analysis already predicted a possible functional divergence based on the reduction in the percentage of amino acid identity, but not similarity, between the two viral proteins. Different reasons could be considered to explain this. A possibility is that, as a consequence of the continuous arms race viruses have with the host immune system, and to escape from the action of the original vCD48, *Saimiri boliviensis* 2B4 diverged. This would imply a loss of the functional restrictions of the S30 gene, which would have mutated substantially and acquired new functions. As a matter of fact, pathogens are major inducers of the evolution of genes involved in the immune response [61,62]. Alternatively, the presence in SMCMV of additional viral products interfering with the CD48:2B4 axis could have made the conservation of S30′s capacity to interact with 2B4 redundant. In this connection, we have reported that murine CMV encodes a viral protein, m154, that reduces CD48 levels at the cell surface of infected cells, and showed in a mouse model that the viral protein promotes viral growth by subverting NK cell responses [63,64]. Similarly, we found that in HCMV-infected macrophages, CD48 is downregulated [65], although in this case, the HCMV protein involved and the consequences of these effects remain to be defined. Therefore, it appears that different CMVs have adopted distinct immunoevasion mechanisms to target 2B4 functions.

In summary, our work shows how the scaffold of CD48, a host immune receptor of the Ig family, can be employed by viruses as an adaptable mold to give rise through evolution to a new family of immune modulators with unique properties. The study of these vCD48s should expand our knowledge of the repertoire of strategies that large DNA viruses exploit to counteract host innate and adaptive immune responses.

## Figures and Tables

**Table 1:** Primer sets used in the study.

Plasmid	Primer	Primer Sequence (5′-3′)
HA-A44	BglIIA44For	AGATCTAATCTTCCTGTTAACGCCATT
	SmaIA44Rev	CCCGGGTCATTTAGCGTACATGTATCC
HA-A45	BglIIA45For	AGATCTGAACCTACACCGAGTCTCTTG
	PstIA45Rev	CTGCAGCTAACGAAAAAATTGTCTCAT
HA-S30	SalIS30For	GTCGACAGACGAATCTATTTATATCGTA
	SalIS30Rev	GTCGACCTATAAACGTTTTCGATAAAC
HA-S31	SalIS31For	GTCGACAATTTTCACATCTCAAGATCCAGATGATTACAGA
	SalIS31Rev	GTCGACCTACGATCGACGTAGCATTCC
A44-Fc	BamHIA44FcFor	GGATCCAAATCTTCCTGTTAACGCCATT
	BamHIA44FcRev	GGATCCACTTACCTGTTTCCATCTTAGTTTGATTACAATTTTC
A45-Fc	BamHIA45FcFor	GGATCCAGAACCTACACCGAGTCTCTT
	BamHIA45FcRev	GGATCCACTTACCTGTATGGTATGTACGTATTGATTC
S30-Fc	BamHIS30FcFor	GGATCCAGACGAATCTATTTATATCGTA
	BamHIS30FcRev	GGATCCACTTACCTGTGTATCGCTGAGAAAGTGGGAC
S31-Fc	BamHIS31FcFor	GGATCCAATTTTCACATCTCAAGATCCAGATGATTACAGA
	BamHIS31FcRev	GGATCCACTTACCTGTTAACGATAATAAGAGGGACGAAGA
Exon2 atCD2	exon2CD2anFor	TTCAGAGATCCTGAAGTAAGC
	exon2CD2anRev	GCTATTTTCCAACTTGCCAAA
Exon3 atCD2	exon3CD2anFor	GTTGCTAGAGGTCTTTGAAATTG
	exon3CD2anRev	GCCTCTGCCTACCAAGGGCCTGAC
Exon2+3 atCD2	SOEx2/3atCD2For	ATATGAAGATTCTAGAGAGAGTCTCAAAAC
	SOEx2/3atCD2Rev	GTTTGAGACTCTCTCTAGAATCTTCATAT
HA-atCD2-Tm	BglIIatCD2TmFor	AGATCTAAAGATGTTAGGAATGCCTTG
	SalIatCD2TmRev	GTCGACACCTTTTTCTGGACAGCTGACGTCCAC
Exon2 atCD58	exon2CD58anFor	GCACATGGTTGGTGCTTCATG
	exon2CD58anRev	CTCTGACAACAGGTAACATTG
Exon3 atCD58	exon3CD58anFor	GCTCAAGGAGTTTGCTCATCG
	exon3CD58anRev	GGGTTTCTGTTAAAAATTGTAACTC
Exon2+3 atCD58	SOEx2/3atCD58For	CTCTTTATGTGCTTGAGCATCTTCCATCTC
	SOEx2/3atCD58Rev	GAGATGGAAGATGCTCAAGCACATAAAGAG
atCD58-Fc	BamHIatCD58For	GGATCCCGGTTCTCAACAAGTTTATGGC
	BamHIatCD58Rev	GGATCCACTTACCTGTCCTTGAATGACCACTGTTTGGGACACAGGT
HA-sb2B4	EcoRIpDFor	GAATTCGGCTTGGGGATATCC
	EcoRIsb2B4pDRev	GAATTCCTGACGGGCATTC
